# Adjustments of selective attention to response conflict – controlling for perceptual conflict, target-distractor identity, and congruency level sequence pertaining to the congruency sequence effect

**DOI:** 10.3758/s13414-021-02294-1

**Published:** 2021-05-04

**Authors:** Miriam Tomat, Mike Wendt, Aquiles Luna-Rodriguez, Thomas Jacobsen

**Affiliations:** 1 Department of Psychology, Experimental Psychology Unit, Helmut-Schmidt-University/University of the Federal Armed Forces Hamburg, Holstenhofweg 85, 22043 Hamburg, Germany; 2 Medical School Hamburg, Hamburg, Germany

**Keywords:** Response conflict, Attentional adjustment, Cognitive and attentional control, Adaptive control, Congruency sequence effect

## Abstract

The congruency sequence effect (CSE) describes the performance difference of congruent trials (in which target and distractor stimuli are associated with the same response) compared to incongruent trials (in which target and distractor stimuli are associated with different responses) as a function of the preceding congruency level (congruent trials relative to incongruent trials). The CSE is commonly interpreted as a measure of conflict-induced attentional adjustment. Although previous research has made substantial progress aiming at controlling for alternative explanations of the CSE, both task-specific and fundamental confounds have remained. In the current study, we used a temporal flanker task, in which two stimuli (i.e., distractor and target) are presented in rapid succession, and extended previous demonstrations of a CSE in flanker tasks by deconfounding target-distractor congruency from perceptual similarity. Using a four-choice task, we could also control for the reversal of distractor-response priming after incongruent trials (which is only feasible in two-choice tasks). Furthermore, we controlled for all confounds based on the sequence (i.e., repetition versus alternation) of the congruency level – such as feature sequence effects, distractor-response contingency switch costs, or temporal learning – by probing the allocation of attention to the points in time of presentation of the first and the second stimulus of a trial. This was achieved by intermixing trials of a temporal search task. The performance accuracy results in this task were consistent with a stronger attentional bias in favor of the target stimulus’ temporal position after incongruent than after congruent trials.

## Introduction

Goal-directed behavior is thought to be supported by a class of cognitive processes, collectively referred to as attentional control. In the laboratory, attentional control is frequently investigated, employing conflict task protocols. These protocols, such as the Eriksen flanker task (Eriksen & Eriksen, 1974), require the participants to respond to task-relevant stimulus features while ignoring task-irrelevant stimulus features. In this task, the participants are instructed to respond to a stimulus presented centrally (i.e., *target*) surrounded by peripheral stimuli (i.e., *distractors)* which are either assigned to the same response as the target (i.e., *congruent* trials) or are assigned to a different response (i.e., *incongruent* trials). Analyzing performance in these protocols typically reveals that responding in congruent trials is, on average, faster and less error-prone than responding in incongruent trials. The *Congruency Effect* (i.e., the performance difference in congruent and incongruent trials) is broadly considered a measure of the influence of task-irrelevant stimulus features on performance. Specifically, it has been attributed to the co-activation of the distractor-related response in addition to the target-related response. Assuming that target- and distractor-related responses compete for response selection and execution, such co-activation should result in a (*response) conflict* in incongruent trials (Eriksen & Eriksen, 1974; for an overview, see Eriksen, 1995). Evidence for distractor-induced response activation has, for instance, been found in reach tracking studies in which participants are instructed to make choice responses by moving their hand to one of several target locations. In such studies, prolonged reach curvatures (diverted trajectories towards the distractor-related response’s target position) in incongruent relative to congruent trials have frequently been observed (e.g., Erb et al., 2016; Erb & Marcovitch, 2018; Scherbaum et al., 2010; Scherbaum & Dshemuchadse, 2019).

Analyzing trial sequences in conflict tasks, a large number of studies found that the congruency level of the predecessor trial modulates the *congruency effect*. Typically, the congruency effect decreases following an incongruent trial relative to following a congruent trial (i.e., *congruency sequence effect,* CSE; e.g., Gratton et al., 1992; Schmidt & Weissman, 2014; Weissman et al., 2014; Weissman et al., 2015). Consistent with the notion that the congruency effect is mainly brought about by conflict in incongruent trials, these findings suggest that the degree of conflict in incongruent trials is lower after incongruent than after congruent predecessor trials. This modulation of the degree of conflict is commonly interpreted as evidence for enhanced attentional focusing induced by conflict. According to the *conflict monitoring account* (Botvinick et al., 2001; Botvinick et al., 2004), a monitoring unit computes each trial's conflict level. A higher level of conflict is supposed to lead to a higher degree of processing selectivity via attentional adjustment (i.e., increasing or decreasing the bias in attentional weights regarding processing the target or distractor) compared to a low level of conflict.

Over the years, it has become increasingly clear that the CSE is subject to various confounding factors, which motivated alternative, non-attentional interpretations of the CSE. Although some of these confounds have been thoroughly discussed in the literature, and efficient methods of controlling them have been proposed (see Braem et al., 2019, for an overview), other confounds are still widely neglected in current experimental studies. In the following, we will first present a brief overview of the former and their remedies and then address two less prominent confounds, one of them being confined to a particular (albeit widely used) kind of experimental task, the other one being inherent in all CSE procedures.

In standard conflict-task protocols, congruency level repetitions tend to involve a larger proportion of trials associated with a complete repetition or a complete alternation of all discriminative stimulus and response features than congruency level alternations, which, in turn, tend to involve a larger proportion of partial repetitions (i.e., repetition of one stimulus or response feature and alternation of another one). Given that partial repetitions are likely to induce interference with memory episodes of previous trials – as laid out in detail in Hommel’s feature integration account (e.g., Hommel et al., 2004) – obtaining a CSE in such conditions may be unrelated to attentional adjustment. This confound has frequently been controlled by a minimum set of four stimulus objects, used as targets and distractors, mapped to four different responses and excluding all trials involving any repetition of a discriminative stimulus or response feature from the analyses. Although the application of this procedure usually failed to eliminate the CSE (e.g., Akçay & Hazeltine, 2011; Bugg, 2008; Hazeltine et al., 2011; Kunde & Wühr, 2006; Purmann et al., 2009; Tomat et al., 2020; Ullsperger, Bylsma, & Botvinick, 2005), using a larger set of stimuli and responses than in the initial CSE studies (e.g., Gratton et al., 1992; Botvinick et al., 1999) raised another problem. As unconstrained (random) stimulus selection might produce only small amounts of congruent trials (i.e., an expected value of 25% in a task involving a 4:4 S-R mapping), researchers tended to bias stimulus selection in favor of congruent trials to produce a more balanced distribution of congruency levels (and of congruency level sequences). Biasing stimulus selection, however, introduces different distractor-related contingencies for congruent and incongruent stimuli. Precisely, individual distractors, occurring more frequently in congruent than in incongruent trials, coincide more often with the “congruent response” (i.e., the response required if the distractor stimulus was presented as target) than with the other (“incongruent”) responses (Schmidt & de Houwer, 2011). Consequently, distractors presented in congruent trials are more predictive of the correct response compared to distractors presented in incongruent trials. Associative learning of these contingencies should facilitate high contingent/congruent trials relative to low contingent/incongruent trials. This assumption has been supported by a corresponding contingency effect obtained with neutral distractors (i.e., stimuli used as distractors that are not assigned a response and never occur as targets). Specifically, Schmidt et al. (2007) administered a variant of the Stroop task, using four different colors as targets and four neutral words, such as *move*, each of which was predominantly presented with one of the colors (i.e., high contingency) and only rarely with each of the other colors (i.e., low contingency). Of importance for the interpretation of the CSE, Schmidt et al. (2007) not only observed faster responses for the high-contingency stimuli than for the low-contingency stimuli but also a reduction of this contingency effect after low-contingency trials compared to after high-contingency trials. Given the confound of contingency levels and congruency levels in studies using non-neutral distractors and biased stimulus selection laid out above, this contingency switch cost allows accounting for the CSE under such circumstances as a modulation of associative priming.

To achieve a 50%/50% ratio of congruent and incongruent trials while controlling for feature sequence and distractor contingency effects, one might use a recently established procedure, which we refer to as the *split-task method*. For this purpose, a four-choice task is divided into two two-choice tasks involving distinct sets of targets and distractors. With this arrangement, contingencies are unbiased when congruent and incongruent trials are administered with a frequency ratio of 50%/50%. Moreover, trial-to-trial feature repetitions can be controlled for by preventing the presentation of the same task in consecutive trials or by confining the analysis to task alternation trials. Several studies reported a CSE with such a task protocol (e.g., Kim & Cho, 2014; Schmidt & Weissman, 2014; Weissman et al., 2014; Weissman et al., 2015). Yet, some experiments using the split-task method failed to obtain a significant CSE (e.g., Weissman et al., 2014), suggesting that at least part of the CSE observed under less controlled conditions is based on confounding factors, leaving only a small effect when these factors are removed.

Despite this progress made in controlling confounds, significant problems have remained. First, we note that the application of the split-task method – as of two-choice tasks in general – might allow for a particular processing strategy that could explain the CSE without reference to attentional adjustment. Specifically, it is conceivable that participants use the distractor to prepare the opposite response than the one the distractor is associated with by instruction (i.e., the response the distractor would require if it were presented as target) after incongruent trials. We refer to this strategy as *reversed distractor-response priming* (see Wühr & Kunde, 2008, for a discussion of this idea in the context of anticipatory conflict control). Preventing this strategy by using four-choice tasks with random stimulus selection has yielded mixed results. Whereas two studies failed to observe a CSE when data from trials associated with repetitions were excluded from the analyses (Mordkoff, 2012; Schmidt & De Houwer, 2011), Tomat et al. (2020) observed a CSE under such conditions in a temporal flanker task, which involves the consecutive presentation of a distractor and a target (Hazeltine et al., 2011). Noteworthy, however, resembling the vast majority of studies using spatial flanker tasks to investigate the CSE (e.g., Gratton et al., 1992; Schmidt & De Houwer, 2011), their stimulus material consisted of perceptually identical objects (i.e., the letters A, B, C, and D) used as targets and flankers. This property of the procedure results in yet another ambiguity regarding the CSE interpretation, namely a confound of congruency level and perceptual target-distractor identity. Under such conditions, it is conceivable that the CSE is brought about by advantageous perceptual segregation of the target and the distractor after a trial involving the same congruency level, hence the same perceptual requirement. This argument might not be confined to the case of target-distractor conjunctions involving identical elements but generalize to cases in which the targets and distractors are perceptually more similar in congruent than in incongruent trials, a frequent property of priming protocols used to investigate the CSE (e.g., Schmidt & Weissman, 2014; Weissman et al., 2014).

Congruency level and perceptual stimulus identity of target-distractor pairs are naturally deconfounded in Stroop-like tasks, that is, in tasks, in which targets and distractors belong to perceptually different stimulus categories such as colors and words for which no systematic relationship of perceptual similarity has been established. Although Blais et al. (2014) reported a CSE in a Stroop task while controlling for stimulus feature sequences and distractor-response contingencies (using a 4:4 S-R mapping and varying the Proportion Congruency conditions), Schmidt (2014) criticized accounting for these results in terms of attentional adjustment based on concerns regarding statistical power and transfer of biased contingencies between blocks of trials. (This criticism might also apply to the study of Tomat et al., 2020.) Moreover, it has recently been suggested that the CSE in the Stroop task is brought about by a different (priming) mechanism than in other conflict protocols, such as flanker and Simon tasks (Aschenbrenner & Balota, 2017), raising doubts about reliance on Stroop task data as the sole source of CSE theorizing.

In light of these developments, further investigation of the CSE, deconfounding congruency, and target-distractor similarity in Stroop tasks or non-Stroop tasks, seems a valuable endeavor. Prime-probe procedures like the temporal flanker task may play a prominent role in this for at least two reasons. First, the availability of distractor information before target information might be a relevant precondition for obtaining a robust CSE in confound-minimized procedures (Weissman et al., 2014). Second, manipulating the length of the distractor-target stimulus-onset asynchrony (SOA) may yield results of theoretical interest. In this connection, the finding of a CSE with a prime-probe (i.e., distractor-target) interval of 1000 ms (Weissman et al., 2015) deserves attention. Intriguingly, in this condition, the congruency effect as such was no longer significant, yielding a CSE in the form of a regular congruency effect after congruent trials and a reversed congruency effect after incongruent trials. As the latter cannot be accounted for in terms of attentional focusing (which would result in the absence of a congruency effect, at most), this finding raises new doubts about the attentional adjustment interpretation of the CSE. In fact, the authors favored a (non-attentional) response modulation account, which attributes the CSE to inhibition of response(s) activated by the distractor, assuming that this inhibition is more efficient if it was involved in the preceding trial. Consequently, the response activated by the distracter is assumed to be inhibited more efficiently if the preceding trial was incongruent compared to if the preceding trial was congruent (cf. Ridderinkhof, 2002). However, the reversal of the congruency effect after incongruent trials might also reflect reversed distractor-response priming after conflict or perceptual facilitation in congruency level repetition trials, as it occurred under conditions of a two-choice task and higher perceptual target-distractor similarity in congruent than in incongruent trials. Nevertheless, the possibility of investigating the CSE in the absence of a congruency main effect offers the possibility of critically testing the attentional adjustment account. Considering the fact that the congruency effect in the temporal flanker task tends to decrease strongly even in four-choice tasks when the prime-probe interval increases (Gillich et al., 2019), this procedure could likely provide such a condition.

At this point, we would like to point out that all the CSE-related confounds hitherto mentioned are a consequence of the fact that the presumed attentional adjustment is assessed in terms of a congruency level repetition advantage. This might allow for more non-attentional explanations than currently identified. In Experiment 3 of the current study, we turn to a new method of assessing conflict-induced attentional adjustment, which arguably controls for all confounds associated with the sequence of congruency levels (cf. Tomat et al., 2020; Wendt et al., 2012; Wendt et al., 2014). As this method has its difficulties – which we discuss in the introduction of Experiment 3 – we first present two conventional CSE experiments in which we examined the CSE in the temporal flanker task under conditions of control of feature sequences, distractor-related contingencies, and perceptual target-distractor similarity. This was achieved by applying an 8:4 S-R mapping in a temporal flanker task. More precisely, the digits 1, 2, 3, and 4, as well as the letters A, B, C, and D were used as stimuli, and digit-letter pairs (i.e., 1-A, 2-B, 3-C, and 4-D) were assigned to four different response keys. Because on each trial, the target and the distractor were drawn from different stimulus categories (i.e., letters vs. digits), congruent trials never involved perceptually identical stimuli.

While preventing perceptually identical target-distractor pairs, drawing targets and distractors from separate stimulus categories also introduced a new possibility of conflict adjustment. Specifically, rather than modulation of the processing of stimulus information presented at a particular point in time, processing adjustment might also be targeted at the stimulus categories (i.e., amplification of the stimulus category from which the target was drawn and/or inhibition of the stimulus category from which the distractor was drawn). Although such adjustment should reveal itself in a CSE if the assignment of target and distractor to the stimulus categories repeats from the preceding trial (e.g., target: digit/distractor: letter ➔ target: digit/distractor: letter), in case the assignment is switched (e.g., target: letter/distractor: digit ➔ target: digit/distractor: letter), performance should be generally impaired after conflict by inhibited processing of the target or increased competition from the distractor. This hypothesized pattern of results is reminiscent of findings observed in task-switching experiments. Task-switching experiments (for an overview, see, e.g., Kiesel et al., 2010; Monsell et al. 2003; Vandierendonck et al., 2010) require participants to execute different tasks in varying sequences, allowing the classification of a given trial as a task repetition (i.e., the same task was executed on the directly preceding trial) or a task switch (i.e., a different task was executed in the directly preceding trial).^1^ In addition to task-switch costs (i.e., impaired performance in task switch trials relative to task repetition trials), task switching experiments have also demonstrated competition between the currently relevant stimulus-response rules and the currently irrelevant task. Specifically, using the same set of motor responses for both tasks, performance tends to be better in trials, in which the same response is called for by both tasks (i.e., congruent trials), than in trials, in which the tasks call for different responses (i.e., incongruent trials). Consistent with the notion of conflict-induced biasing of task readiness (i.e., amplification of the relevant task’s mental set and/or inhibition of the competitor task’s mental set in incongruent trials), analyzing task performance as a function of the congruency level of the preceding trial demonstrated a CSE when the task repeated but not when the task alternated (Brown et al., 2007; Kiesel et al., 2006; Schneider, 2015; Wendt et al., 2013), as well as larger task switch costs after incongruent, compared to congruent trials (Brown et al., 2007; Goschke, 2000; Kiesel et al., 2006; Schneider, 2015; Wendt et al., 2013).

In light of these previously observed effects in task-switching studies and our considerations concerning the conflict-induced adjustment of target and distractor categories, it seems useful to analyze the CSE as a function of repetition versus alternation of the sequence of stimulus categories (i.e., digit first/letter second or letter first/digit second) or, if conceived of as distinct (digit and letter identification) tasks, of task repetition versus alternation. Whereas adjustment concerning the processing of stimuli presented at particular points in time within a trial (temporal attentional adjustment; i.e., amplification of processing the second [target] stimulus and/or inhibition of processing of the first [distractor] stimulus after conflict) should be effective for both repetition and alternation trials, thus eliciting a CSE in both cases, adjustment concerning the processing of category-specific stimuli (i.e., digits, letters) or task-sets (digit identification, letter identification) should not. More precisely, inhibition of the distractor category or amplification of the target category should only reduce the distractor's influence on performance (thus producing a CSE) if the assignment of stimulus categories to the target and the distractor is maintained in the subsequent trial. By contrast, switching this assignment would require responding to a stimulus of the previously inhibited category and/or suffering interference from a stimulus or the previously amplified category. Thus, we would expect a CSE in repetition trials and performance decrement in alternation trials after an incongruent compared to after a congruent stimulus.

In summary, despite almost three decades of research on the CSE in flanker tasks, unequivocal evidence for increased attentional focusing on target-related information, evoked by response conflict, is still lacking. In Experiments 1 and 2 of the current study, we controlled for previously identified confounds with stimulus and response feature sequences, distractor-response contingencies, and perceptual target-distractor similarity, preventing a strategy of conflict-induced reversal of distractor-response priming, by use of a temporal flanker task protocol including eight stimuli (four digits and four letters), mapped onto four responses.

## Experiment 1

### Method

#### Participants

Sixteen healthy students (seven females and nine males) ranging in age from 22 to 46 years of the University of Hamburg gave informed consent to participate in a single-session study in exchange for partial fulfillment of course requirements.

#### Stimuli

The stimulus material for the temporal flanker task was the letter-digit pairs. Each of the letters *A*, *B*, *C*, and *D*, and the digits *1*, *2*, *3*, and *4*, could occur as a target or distractor stimulus. The characters measures were horizontally 8 mm × 13 mm vertically., All stimuli were presented in the center of the screen, in white color on a grey background in the center of a white frame (41 mm × 43 mm). The frame was presented continuously.

#### Procedure

**Fig. 1:**
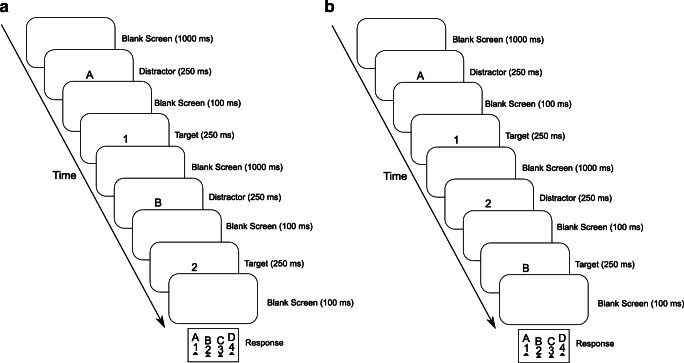
Schematic diagram of two (congruent) consecutive temporal flanker task trials involving the successive presentation of a distractor letter and a target digit with a repetition of the stimulus category assignment of target and distractor (**A**) and an alternation of the stimulus category assignment of target and distractor (**B**) of Experiment 1

Participants viewed the screen (refresh rate of 60 Hz) from a distance of about 70 cm. The temporal flanker task trials consisted of a blank screen for 1000 ms, followed by a distractor for 250 ms, then a blank screen for 100 ms, followed by the target for 250 ms, and a blank screen until response. Following an incorrect response, a message “falsch” (German for incorrect) was presented for 800 ms right below the stimulus frame. The target-distractor pair of the trial with the wrong response was repeated but was not recorded as a trial. For every trial, distractor and target were chosen randomly with the constrain that distractor and target were a digit and a letter or vice versa. Responses were collected using a purpose-built keyboard (response-time resolution < 1 ms). The letters were mapped onto the response keys in alphabetical order from left to right, and the digits were mapped onto the response keys in numerical order from left to right. Participants pressed the four lateral keys with the middle and index fingers of their left and right hands and were instructed to rest their fingers on the keys between trials. Blocks of 99 trials were presented, starting with a practice block followed by overall 24 experimental blocks with 99 trials per block. After the first 13 blocks, the participants took a break for about 15 min. For a schematic diagram of a temporal flanker task trial, see Fig. 1.

#### Data analysis

We excluded data from trials associated with reaction times (RTs) below 200 ms or above 2500 ms in the following analyses. Only data from trials involving a correct response were subjected to the RT analyses. Finally, the analyses were confined to data from trials devoid of feature repetitions and repetitions of the assigned response. On average, we excluded 51% of trials per subject for the RT analyses of Experiment 1. We also excluded data from the first three trials per block, from the practice block, and from trials following an error. Thereof one congruent trial was excluded due to the RT restriction criteria. (for the results, including feature repetitions and repetitions of the assigned response, see Appendix A). We filtered the data with the *tidyr* (Wickham & Henry, 2019) and dplyr (Wickham et al., 2020) package, and analyzed it with the *ez* package (Lawrence & Lawrence, 2016) implemented in R (R Core Team, 2019).

Analyses of Variance (ANOVAs) with repeated measures on the factors Congruency Level of Current Trial (congruent vs. incongruent), Congruency Level of Preceding Trial (congruent_n-1_ vs. incongruent_n-1_), and Sequence of Target/Distractor Category (repetition vs. alternation) were conducted on the RTs and error proportions. Note that we report the results of one-tailed significance tests due to our directional hypotheses.

### Results

#### RT

**Fig. 2:**
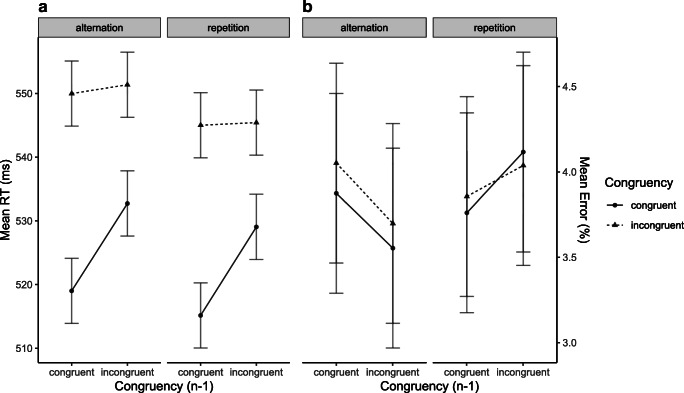
Mean reaction times (RTs) (**A**) and mean error proportions (**B**) of temporal flanker task trials in Experiment 1. Data are shown as a function of Congruency Level of Current Trial (congruent vs. incongruent), Congruency Level of Preceding Trial (congruent_n-1_ vs. incongruent_n-1_), and Sequence of Target/Distractor Category (alternation vs. repetition)

The ANOVA overall revealed a significant main effect of Congruency Level of Current Trial (*F*(1, 15) = 16.36, *p* < .001, *η*_*p*_^2^ = .52), reflecting larger RTs in incongruent than in congruent trials. The analysis also revealed that both main effects reached significance (Congruency Level of Preceding Trial (*F*(1, 15) = 5.74, *p* = .015, *η*_*p*_^2^ = .28), reflecting larger RTs after incongruent than after congruent trials; Sequence of Target/Distractor Category (*F*(1, 15) = 7.85, *p* = .0067, *η*_*p*_^2^ = .34, indicating larger RTs when the target and distractor category alternated from the preceding trials than when they repeated. Of most importance, the analysis revealed a significant CSE (*F*(1, 15) = 3.37, *p* = .043, *η*_*p*_^2^ = .18) which was not modulated by Sequence of Target/Distractor Category (*F*(1, 15) = 0.08, *p* = .39, *η*_*p*_^2^ = .0051) (see Fig. 2). The analysis of the trials only involving target/distractor category repetitions did not reveal a significant CSE (*F*(1, 15) = 2.87, *p* = .055, *η*_*p*_^2^ = .16).

#### Error proportion

The ANOVA revealed no significant effects (all *F*s <1; see Fig. 2).

### Discussion

The results of Experiment 1 extended previous findings of a CSE in a temporal flanker task (Hazeltine et al., 2011; Tomat et al., 2020) by deconfounding congruency and target-distractor identity. As a consequence of using an 8:4 mapping, selecting a target and distractor stimulus randomly (from different categories) on each trial, and excluding all data from trials associated with a feature repetition from the analyses, the CSE observed in Experiment 1 can neither be attributed to biased stimulus feature sequences, biased distractor-related contingency sequences, or reversed distractor-response priming after conflict, nor can it be accounted for in terms of stimulus-conflict-evoked attentional adjustment. Notably, the CSE was entirely driven by RT differences in congruent trials, whereas RTs in incongruent trials did seem affected by the preceding trial's congruency level (see Fig. 2). This pattern of the CSE – referred to as *post-conflict slowing* (Verguts et al., 2011) – is not unusual (e.g., Gratton et al., 1992; Lamers & Roelofs, 2011; Ullsperger et al., 2005; Wendt, Kluwe, & Peters, 2006; see also Tomat et al., 2020) and has been suggested to reflect an additional adjustment mechanism of increased response caution after experiencing conflict.

Although the experiment yielded a general cost of switching target and distractor categories, resembling the task switch cost usually observed in task-switching studies, the CSE was not affected by the sequence of the assignment of digit and letter stimuli to the target and the distractor stimulus. As laid out above, this result pattern deviates from previous findings in task-switching studies, in which the CSE was confined to task repetition trials (Brown et al., 2007; Kiesel et al., 2006; Schneider, 2015; Wendt et al., 2013) and suggests an adjustment of temporal attention (i.e., the strength of processing stimuli presented at first vs. second temporal position of stimulus onset in a trial) rather than control operations targeted at task-specific representations, such as digit/letter stimuli categories, as the underlying mechanism of the CSE. Corroborating this view, there was no increase in switch costs after incongruent trials as found in previous task-switching studies, commonly attributed to conflict evoked priming of task-set (Brown et al., 2007; Goschke, 2000; Kiesel et al., 2006; Schneider, 2015; Wendt et al., 2013).

Due to the small sample size in Experiment 1, the test power was low. A post hoc power analysis using R (R Core Team, 2019) with the package pwr (Champely et al., 2015) for one-sample one-tailed t-tests revealed with *n* = 16, a medium effect size (d = .43) and an alpha = .05, a power of .50. Given the comparably small sample size of Experiment 1, we decided to replicate the experiment with increased statistical power^2^.

## Experiment 2

### Method

#### Participants

An a priori power analysis was conducted using R (R Core Team, 2019) with the package pwr (Champely et al., 2015) to test the difference between (the Congruency Effect after congruent and the Congruency Effect after incongruent trials) two dependent group means, using a two-tailed t-test, a medium effect size (d = .5), and an alpha of .05. The result showed that a total sample of 32 participants was required to achieve a power of approximately .80. Therefore, according to a priori power analysis results, 32 healthy students (17 female and 15 male) ranging in age from 19 to 30 years of the Helmut-Schmidt-University/University of the Federal Armed Forces Hamburg gave informed consent to participate in a single-session study in exchange for partial fulfillment of course requirements.

#### Stimuli

Stimulus material was the same as in Experiment 1.

#### Procedure

The procedure was equal to Experiment 1 with a few deviants. In Experiment 2, there was no break of 15 min after the first half of the experimental session. Experiment 2 involved 18 experimental blocks that consisted of 96 trials each, and there was no white frame presented surrounding the stimuli.

#### Data analysis

Data analysis was equal to Experiment 1. On average, we excluded 35% of trials per subject for the RT analyses of Experiment 2. Thereof 57 trials were excluded due to the RT restriction criteria (27 congruent and 30 incongruent trials). For the results, including all feature repetitions, see Appendix B. Note that, in the course of consistency, as in Experiment 1, we report the results of one-tailed significance tests due to our directional hypotheses.

### Results

#### RT

**Fig. 3:**
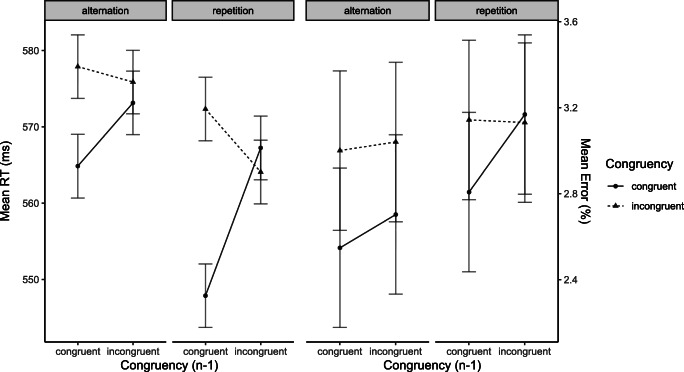
Mean reaction times (RTs) (**A**) and mean error proportions (**B**) of temporal flanker task trials of Experiment 2. Data are shown as a function of Congruency Level of Current Trial (congruent vs. incongruent), Congruency Level of Preceding Trial (congruent_n-1_ vs. incongruent_n-1_), and Sequence of Target/Distractor Category (alternation vs. repetition)

The ANOVA overall revealed a significant main effect of Congruency Level of Current Trial (*F*(1, 31) = 7.27, *p* = .006, *η*_*p*_^2^ = .19), reflecting larger RTs in incongruent than in congruent trials. The analysis also revealed a significant main effect of Congruency Level of Preceding Trial (*F*(1, 31) = 4.21, *p* = .025, *η*_*p*_^2^ = .12), reflecting larger RTs after incongruent than after congruent trials and it also revealed a significant main effect of Sequence of Target/Distractor Category (*F*(1, 31) = 35.02, *p* < .001, *η*_*p*_^2^ = .53), indicating larger RTs when the target and distractor category switched from the preceding trials. Of most importance, the analysis revealed a significant overall CSE (*F*(1, 31) = 20.03, *p* < .001, *η*_*p*_^2^ = .39). This interaction, however, was modulated by Sequence of Target/Distractor Category (*F*(1, 31) = 4.49, *p* = .021, *η*_*p*_^2^ = .13) (see Fig. 3). To clarify this three-way interaction, we conducted additional ANOVAs separately for trials only involving target/distractor category repetitions (which revealed a significant CSE (*F*(1, 31) = 25.85, *p* < .001, *η*_*p*_^2^ = .45), and for trials only involving target/distractor category alternations did not reveal a significant CSE (*F*(1, 31) = 2.68, *p* = .055, *η*_*p*_^2^ = .079).

#### Error proportion

The ANOVA revealed no significant effects (all *F*s <1; illustrated in Fig. 3).

### Discussion

The results of Experiment 2 corroborated the most important result of Experiment 1, namely the occurrence of a CSE notwithstanding the deconfounding of congruency and perceptual target-distractor identity. Despite the use of a similar procedure, however, an interesting discrepancy with the results of Experiment 1 could be observed. Specifically, the CSE was larger in target/distractor category repetition trials than in target/distractor category alternation trials (in which it failed to reach statistical significance). Although it is conceivable that this three-way interaction went undetected in Experiment 1 because of the lower statistical power, it must also be considered that different strategies of adjustment (i.e., attentional weights assigned to the time of target and distractor occurrence vs. to task-specific representations, such as digit/letter stimulus categories) may take place, depending on not yet understood contextual factors or individual preferences. We will come back to this issue in the Discussion of Experiment 3.

Although our Experiments 1 and 2 added new evidence for trial-by-trial attentional adjustment to response conflict, which has so far only been found for the Stroop task (Blais et al., 2014), a more general problem pertaining to the dismissal of non-attentional accounts of the CSE deserves discussion. This problem lies in the fact that controlling for any set of confounds conceivable to facilitate the processing of congruency level repetitions compared to congruency level alternations for reasons other than attentional adjustment might not be sufficient to rule out facilitated processing brought about by the repetition of yet another – not yet identified – abstract feature associated with congruent versus incongruent trials. To illustrate this point, we would like to consider two specific possibilities. First, it is conceivable that the CSE is brought about by enhanced efficiency of conflict resolution after an incongruent trial, rather than by reduced conflict accrual as a result of adjustment of attentional weights. The response modulation account (Ridderinkhof, 2002), mentioned in the Introduction, can be considered an instance of this idea. Contrasting with the reversal of response priming idea, accounts of conflict-induced strengthening of conflict resolution would predict a CSE not only in two-choice tasks.

Second, another powerful account regarding the CSE, not discussed in this article so far, has been referred to as temporal learning (Schmidt, 2013). According to the temporal learning account, the cognitive system learns not only how but also when to respond to a given stimulus. Therefore, performance is facilitated if the preceding trial's response timing matches the current trial's response timing. Given that responding is generally faster in congruent relative to incongruent trials, the CSE might reflect facilitated processing brought about by a closer match of the current trial’s response timing characteristics with expectations based on the previous trial's experience the congruency level repeats.^3^

Given the fact that neither conflict-induced strengthening of conflict resolution nor temporal learning – nor facilitated processing brought about by the repetition of a hitherto unidentified feature which co-varies with the congruency level – can be ruled out through the control measures applied so far, extending the methodological repertoire to assess attentional adjustment beyond CSE examination procedures might constitute a significant improvement. Noteworthy, in this connection, some previous studies involved attempts to manipulate or assess the strength of conflict in a more fine-grained manner. For instance, Forster et al. (2011) varied the number of flanker stimuli that were associated with an incorrect response (and, inversely, the number which was identical with the target) and examined the congruency effect as a function of the, such defined, three different levels of incongruency of the predecessor trial. Similarly, Hubbard et al. (2017), using a conflict task in which a distractor appeared at uncertain locations, assessed the congruency effect in trials following incongruent trials associated with overt fixation of the distractor (assumed to yield larger conflict) versus without such fixation.

Whereas these latter methods extend standard CSE methodology by subdividing incongruent trials used to elicit conflict, there have also been attempts of applying other measures of attentional adjustment than the CSE by administering trials of a probe task, requiring the processing of stimulus information which elicited conflict in the preceding trial. Specifically, Wendt et al. (Wendt et al., 2012, cf. Verguts et al., 2011) investigated attentional adjustment in a spatial flanker task (i.e., a target presented at a central location flanked by two instances of a distractor stimulus, one on each side). Trials could be congruent or incongruent, and the ratio of congruent to incongruent trials was manipulated between different parts of the experimental session. To probe the allocation of visual attention to the locations of the target and the distractors in conditions associated with high and low congruent/incongruent ratios, as well as following a congruent and following an incongruent predecessor trial, trials of a visual search task occurred with the equal likelihood in the to be compared conditions. In a search task trial, three different digits were presented at the same locations as the letters of the flanker task. Participants had to search for a target digit, randomly presented at any of the three locations. Consistent with the notion that frequent conflict, evoked by the flanker stimuli, results in increased narrowing of the focus of visual attention to the central stimulus location, search times displayed a more pronounced center-to-periphery gradient under conditions of a lower congruent/incongruent ratio. By contrast, however, the search time pattern did not differ significantly after congruent and incongruent predecessor trials.

Tomat et al. (2020) modified the search task to investigate attentional adjustment in the temporal flanker task. In this “temporal search task,” two stimuli were presented in close succession (associated with the same timing characteristics as the stimulus presentation in temporal flanker task trials). One of these stimuli presented randomly at the first or the second temporal position acted as the target while the other stimulus did not afford any response (i.e., neutral distractor). Contrasting with the results of the search task obtained in the context of the spatial flanker task employed by Wendt et al. (2012), responding to targets presented at the first versus at the second temporal position was neither significantly affected by the congruency level of the directly preceding flanker task trial nor by the congruent/incongruent ratio in the temporal flanker task. Despite these null findings, we deem further investigations with this methodological approach necessary given the principal problems inherent in inferring attentional adjustments from observing a CSE. Experiment 2 was conducted for this purpose.

## Experiment 3

Experiment 3 involved a combination of trials of a temporal flanker task and trials of a “temporal search task,” replicating the experiment reported in Tomat et al. (2020) with two modifications. First, to alleviate the confound of congruency and perceptual target-distractor similarity, we mapped the same eight stimuli onto the same four responses as in Experiments 1 and 2 of the current study. Second, rather than choosing target and distractor stimuli randomly in each trial, we applied the above-mentioned split-task method, creating two subsets of stimuli with one stimulus set including the characters 1, A, 2, and B, and the other one including the characters 3, C, 4, and D. The letters were mapped onto the response keys in alphabetical order from left to right. The digits were mapped onto the response keys in numerical order from left to right. Consecutive flanker task trials always involved stimuli from different subsets, precluding all stimulus and response repetitions during task administration while allowing us to present a 50:50 ratio of congruent and incongruent trials.

For the temporal search task, the targets and distractors were composed of Landoltrings in different colors, presented at respectively either of the two consecutive temporal positions. Targets and distractors were defined by specific conjunctions of the position of the gap in the ring and stimulus color. Each trial involved a target and a distractor, presented at randomly chosen temporal positions. Attentional adjustment to conflict in the temporal flanker task (i.e., increased [reduced] deployment of temporal attention to the time of target [distractor] presentation) should evidence itself in relatively improved performance for search task targets presented at the second temporal position, compared to search task targets presented at the first temporal position, after incongruent compared to after congruent flanker task trials. We thus predict an interaction of the search task target's temporal position and the congruency level of a directly preceding temporal flanker task trial.

### Method

#### Participants

The sample size was determined by a comparable experiment (main differences to Experiments 1 and 2: four-choice task, confound with perceptual target flanker identity; see Tomat et al., 2020) for which the RT analysis yielded a substantial PCE with 23 participants (*η*_*p*_^2^ = .33). Twenty-four healthy students (15 females and nine males) ranging in age from 21 to 29 years of the Helmut-Schmidt-University/University of the Federal Armed Forces Hamburg gave informed consent to participate in a single-session study in exchange for partial fulfillment of course requirements.

#### Stimuli

Stimulus material for the temporal flanker split-task was again the letter-digit combinations A, 1; B, 2; C, 3; and D, 4. All these stimuli were presented in the screen center, in white color on a grey background. For the temporal search task, Landoltrings of the colors red, yellow, green, and blue were used and presented in the center of the screen on a grey background. The gaps were rotated to the 45°, 135°, 225°, and 315° positions to make them correspond roughly with the positions of the keys on the response keyboard.

#### Procedure

**Fig. 4:**
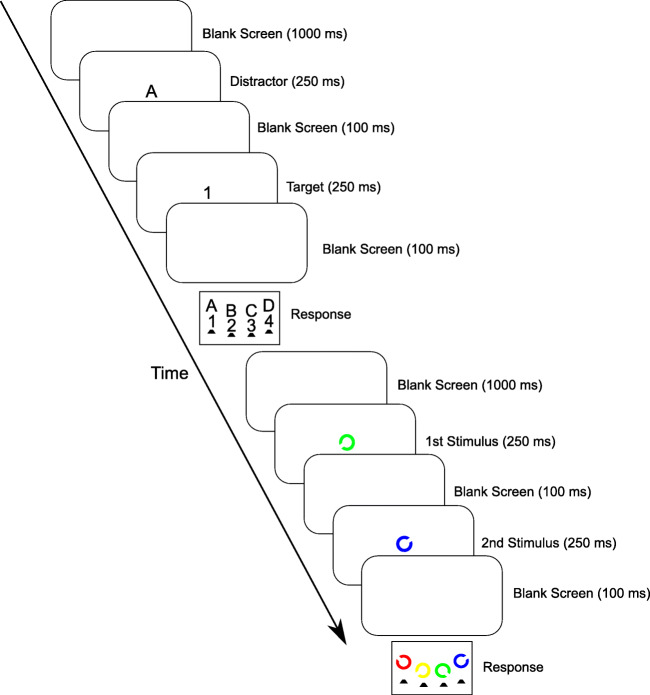
Schematic diagram of a trial sequence of a preceding (congruent) temporal flanker task trial succeeded by a temporal search task trial involving the successive presentation of a distractor (a Landoltring) on first and a target (a Landoltring) on second temporal position of Experiment 3

The S-R mapping was identical to that in Experiments 1 and 2. Each temporal flanker split-task trial (temporal flanker task; Hazeltine et al., 2011) involved the consecutive presentation of a distractor letter/digit and a target letter/digit, each shown for 250 ms and separated by a 100 ms blank screen. Participants were instructed to respond exclusively to the stimulus on the second temporal position. Consecutive trials always involved alternation of the stimulus subsets described above. The temporal search task had an occurrence probability of 50% following a temporal flanker task trial and of 0% following a temporal search task trial, resulting in an overall 33% probability of occurrence. On each trial of the temporal search task, two rings were presented consecutively with the same temporal presentation characteristics as in the temporal flanker task. The temporal position of the target was chosen randomly. Targets and distractors were defined by the conjunction of the ring’s aperture position and color. The conjunctions “top-left/red,” “top-right/blue,” “bottom-left/yellow,” and “bottom-right/green” acted as targets, requiring the response that corresponded spatially with the location of the ring’s gap. The other twelve possible conjunctions of the four colors and the four gap locations were used as distractors. The target and the distractor were chosen randomly from the respective sets of stimuli on each temporal search task trial. Participants were instructed to respond exclusively to the target. Response device and stimulus-response assignment for the temporal flanker task was identical to Experiments 1 and 2. The targets Landoltring aperture and color combinations were mapped onto the response keys in the order upper-left: red, upper-right: blue, lower-left: yellow, and lower-right: green. Participants pressed the four lateral keys with the middle and index fingers of their left and right hands and were instructed to rest their fingers on the keys between trials. The trial structure was identical to Experiments 1 and 2. The same structure held for the temporal search task, except the target/distractor, could occur at the first or the second temporal position. Blocks of 99 trials were presented, starting with a practice block for each task separately and a practice block with both tasks mixed, followed by 12 experimental blocks with 99 trials per block. Figure 4 depicts a schematic diagram of a temporal flanker task trial followed by a temporal search task trial.

#### Data analysis

The criteria for trial exclusion were identical to Experiments 1 and 2. On average, we excluded 32% of trials per subject for Experiment 2. Thereof no trial was excluded due to the RT restriction criteria. Only data from trials in which the correct response was given were subjected to the RT analyses. We filtered the data with the *tidyr* (Wickham & Henry, 2019) and dplyr (Wickham et al., 2020) package, and analyzed it with the *ez* package (Lawrence & Lawrence, 2016) implemented in R (R Core Team, 2019). We excluded the data of one participant due to a lack of valid data in one condition for all analyses.

For the temporal flanker task, the analyses were identical to Experiments 1 and 2. These analyses were confined to trials preceded by another temporal flanker task trial. Additional ANOVAs were conducted on repetition and alternation of Sequence of Target/Distractor Category trials mean RTs and error proportions separately. In light of the fact mentioned in Footnote 2, that Erb and Aschenbrenner (2019) observed a CSE selectively in sequences in which the congruency level of trial N-1 matched the level of trial N-2, we conducted additional analyses including the factor Preceding Trial’s Congruency Level Sequence (repetition vs. alternation). These analyses are reported in Appendix C. For the temporal search task, ANOVAs with repeated measures on the factors Congruency Level of Preceding Trial (congruent_n-1_ vs. incongruent_n-1_) and Temporal Target Position (first vs. second) were conducted on the RTs and error proportions. Again, we report the results of one-tailed significance tests due to our directional hypotheses.

### Results

#### Temporal flanker task

##### RT

**Fig. 5:**
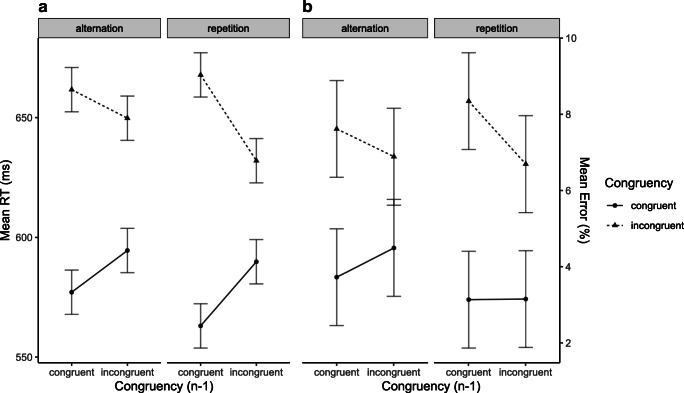
Mean reaction times (RTs) (**A**) and mean error proportions (**B**) of temporal flanker task trials in Experiment 3. Data are shown as a function of Congruency Level of Current Trial (congruent vs. incongruent), Congruency Level of Preceding Trial (congruent_n-1_ vs. incongruent_n-1_), and Sequence of Target/Distractor Category (alternation vs. repetition)

The ANOVA overall revealed a significant main effect of Congruency Level of Current Trial (*F*(1, 22) = 43.83, *p* < .001, *η*_*p*_^2^ = .7), and a marginally significant main effect of Sequence of Target/Distractor Category (*F*(1, 22) = 2.0, *p* = .08, *η*_*p*_^2^ = .08). Furthermore, the results showed a significant CSE (*F*(1, 22) = 14.18, *p* < .001, *η*_*p*_^2^ = .4) which was also modulated by Sequence of Target/Distractor Category (*F*(1, 22) = 3.48, *p* = .037, *η*_*p*_^2^ = .1) (see Fig. 5). No other effects reached significance. The analysis for the trials only involving target/distractor category repetitions revealed a significant main effect for Congruency Level of Current Trial (*F*(1, 22) = 28.29, *p* < .001, *η*_*p*_^2^ = .6), reflecting larger RTs in incongruent than in congruent trials. More importantly, it also revealed a significant CSE (*F*(1, 22) = 16.27, *p* < .001, *η*_*p*_^2^ = .4 ). The analysis for the trials only involving target/distractor category alternations revealed a significant Congruency Effect (*F*(1, 22) = 41.14, *p* < .001, *η*_*p*_^2^ = .7), and a significant CSE (*F*(1, 22) = 3.98, *p* = .03, *η*_*p*_^2^ = .2).

##### Error proportion

The ANOVA overall revealed a significant main effect of Congruency Level of Current Trial (*F*(1, 22) = 18.88, *p* < .001, *η*_*p*_^2^ = .5) and a significant CSE (*F*(1, 22) = 3.01, *p* = .045, *η*_*p*_^2^ = .1). We also found a marginally significant two-way interaction of Congruency Level of Current Trial and Sequence of Target/Distractor Category (*F*(1, 22) = 2.26, *p* = .07, *η*_*p*_^2^ = .1) (see Fig. 5). No other effects reached significance. The analysis for the trials only involving target/distractor category repetitions only revealed a significant main effect for Congruency Level of Current Trial (*F*(1, 22) = 25.66, *p <* .001, *η*_*p*_^2^ = .5). The analysis of the trials only involving target/distractor category alternations revealed a significant Congruency Effect (*F*(1, 22) = 9.08, *p* = .003, *η*_*p*_^2^ = .3).

#### Temporal search task

##### RT

**Fig. 6:**
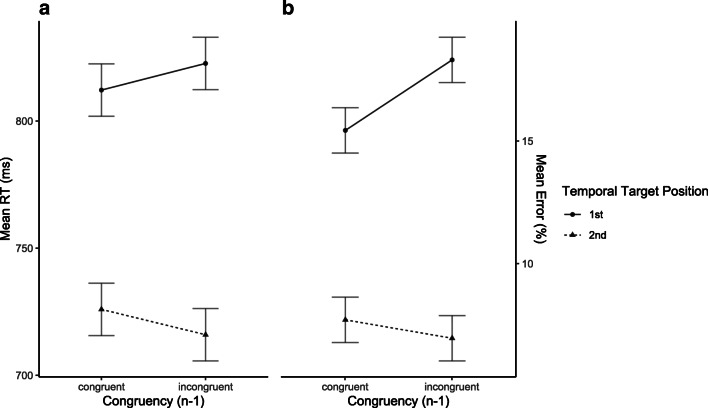
Mean reaction times (RTs) (**A**) and mean error proportions (**B**) of temporal search task trials with preceding temporal flanker task trials in Experiment 2. Data are shown as a function of Temporal Target Position (first vs. second) and Congruency Level of Preceding Trial (congruent_n-1_ vs. incongruent_n-1_)

The ANOVA revealed a significant main effect of Temporal Target Position (*F*(1, 22) = 7.68, *p* = .005, *η*_*p*_^2^ = .3), reflecting larger RTs for responses to targets presented on the first compared to the second temporal position. There was a marginally significant interaction of Temporal Target Position and Congruency Level of Previous Trial (*F*(1, 22) = 2.1, *p* = .08, *η*_*p*_^2^ = .1), but no significant main effect of Congruency Level of Previous Trial (*F*(1, 22) = .002, *p* = .48, *η*_*p*_^2^ < .001) (see Fig. 6).

##### Error proportion

The ANOVA revealed a significant main effect of Temporal Target Position (*F*(1, 22) = 21.31, *p* < .001, *η*_*p*_^2^ = .5), reflecting that more errors were made to targets presented on the first compared to the second temporal position. Most importantly, error proportions of responses to targets presented on the first temporal position but not concerning responses made to targets presented on the second temporal position were increased after incongruent compared to after congruent flanker task trials, yielding the predicted significant two-way interaction of Temporal Target Position and Congruency Level of Previous Trial (*F*(1, 22) = 8.28, *p* = .004, *η*_*p*_^2^ = .3, see Fig. 5). The main effect of Congruency Level of Previous Trial was only marginally significant (*F*(1, 22) = 2.62, *p* = .06, *η*_*p*_^2^ = .1).^4^

### Discussion

Similar to the results of Experiment 2, the CSE was less pronounced in target/distractor category alternation trials than in target/distractor category repetition trials. The most important novel aspect of Experiment 3, however, pertains to the occasional occurrence of the temporal search task, in which the target randomly occurred in the first or in the second temporal position. Performance in the temporal search task was characterized by the predicted interaction, that is, by relatively improved responding to targets presented at the second temporal position (relative to targets presented at the first temporal position) after incongruent compared to after congruent predecessor trials, albeit the effect was significant only in the error analysis. A possible way to reconcile this evidence for adjustment of temporal attention with the lack thereof in the temporal flanker task (and in the search task results of Tomat et al., 2020) lies in the assumption of context-dependency of the adjustment mechanism. Evidence for such context-dependency has been found by varying a task-irrelevant context feature and observing the CSE only in trials associated with a repetition of that feature (Spapé & Hommel, 2008). Although an interpretation of the CSE, in that case, is difficult as no confound-minimized procedure was applied, assuming that the perception of specific contextual changes between consecutive trials substantially reduces the likelihood of attentional adjustment to occur, intermixed trials of another task involving a distinct set of stimuli are liable to lack such adjustment.

## General discussion

The current study aimed at controlling widespread confounds in investigations of conflict-induced attentional adjustment. In a series of three experiments, in which target and distractor were drawn from different stimulus categories in each trial and the trial-to-trial sequence of congruency levels was deconfounded from the sequences of basic stimulus and response features and distractor-related contingencies, we consistently observed a CSE (less pronounced when target/distractor category alternated relative to when it repeated), ruling out explanations in terms of conflict at the level of stimulus representations rather than responses as the cause of the CSE. Moreover, intermixing trials of a temporal search task yielded a significant interaction of the temporal position of the search task target and the congruency level of a preceding temporal flanker task trial, that is, a larger advantage of response accuracy for targets presented at the second position, compared to targets presented at the first position, after incongruent than after congruent trials. To our knowledge, this is the first piece of behavioral evidence for trial-to-trial conflict-induced attentional adjustment beyond the observation of a CSE. The fact that the latter effect was only marginally significant in the RT analysis far from significance in the study of Tomat et al. (2020), which involved a very similar procedure to that in Experiment 3 of the current study, suggests that deriving useful probe task measures for the assessment of attentional deployment depends on meeting constraints not well understood so far. Although the development of more sensitive probe task techniques may be a tedious endeavor, it seems a valuable if not required route to take not only for the sake of corroborating our preliminary evidence extending CSE measures but also concerning achieving a better understanding of the role of the sequence of contextual factors of the task environment in conflict-induced attentional adjustment.

Regarding the temporal flanker task, it is noteworthy that apart from a robust CSE in trials associated with a repetition of the assignment of stimulus categories to the target and the distractor, effects of relevance concerning the adjustment mechanism appeared remarkably variable. First, consistent with occasional occurrence in previous studies investigating the CSE, post-conflict slowing, that is, slower responding in trials following an incongruent compared to a congruent predecessor trial, was observed in Experiment 1 but not in Experiments 2 and 3. Reach tracking studies investigating cognitive control might offer a potential explanation for the elusive occurrence of this effect. Recent studies attempted to measure two processes underlying cognitive control separately – a response threshold adjustment process and a response selection process – by tracking motor response initiation times and reach curvatures (e.g., Erb et al., 2016). They found post-conflict slowing only in response initiation times but not in reach curvatures (Erb et al., 2016; Erb & Marcovitch, 2018). It is conceivable that standard protocols relying on RTs of key-press responses tend to mask the slowing of response initiation times.

Second, the CSE in trials associated with a switch of the assignment of stimulus categories to the target and the distractor seemed less pronounced, yielding significant three-way interactions of congruency in the current and in the previous trial and the sequence of the assignment in Experiments 2 and 3. This pattern of findings is consistent with either context-dependency of adjustment of temporal attention (assuming that the order of presenting digit and letter stimuli constitutes an effective context) or with (additional) adjustment operations applied to the processing of individual stimulus categories or category-specific S-R translation processes. In this connection, the possibility of multiple adjustment strategies, depending on the specific task demands or on individual representations of the task environment, must be considered. Future research of conflict-related attentional adjustment might proceed in identifying such strategies in more detail. Although including target/distractor category sequence in the analyses of the experiments reported in the current article was but a by-product of the experimental set-up we used to achieve an 8:4 stimulus-response mapping of stimuli to responses – resulting from an attempt to minimize memory load by employing well-known stimulus categories involving for items each in familiar left-to-right order – a more systematic investigation of factors associated with different patterns of the CSE in trials associated with repetition and alternation might be a helpful tool in this process.

## Figures and Tables

**Fig. 7:**
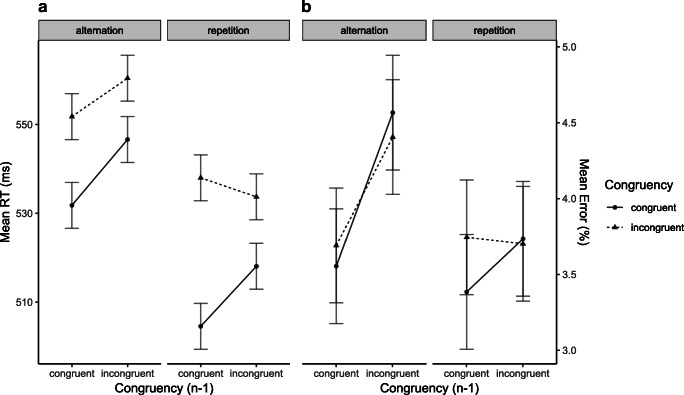
Mean reaction times (RTs) (**A**) and mean error proportions (**B**) of temporal flanker task trials in Experiment 1 involving all feature repetitions. Data are shown as a function of Congruency Level of Current Trial, Congruency Level of Preceding Trial, and Sequence of Target/Distractor Category (alternation, repetition)

**Fig. 8:**
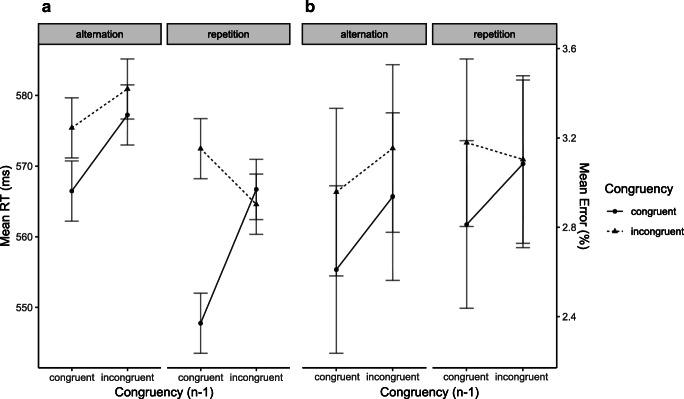
Mean reaction times (RTs) (**A**) and mean error proportions (**B**) of temporal flanker task trials of Experiment 2 involving all feature repetitions. Data are shown as a function of Congruency Level of Current Trial, Congruency Level of Preceding Trial, and Sequence of Target/Distractor Category (alternation, repetition)

**Fig. 9:**
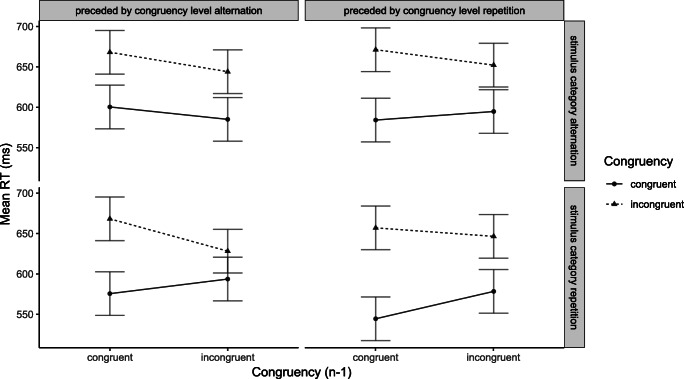
Mean reaction times (RTs) of temporal flanker task trials in Experiment 3 only involving temporal flanker task trials. Data are shown as a function of Congruency Level of Current Trial, Congruency Level of Preceding Trial, Sequence of Target/Distractor Category (alternation, repetition), and Preceding Trial’s Congruency Level Repetition Type (alternation, repetition)

**Fig. 10:**
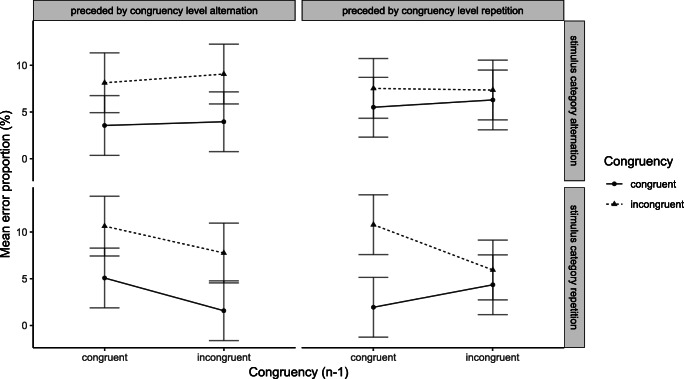
Mean error proportions of temporal flanker task trials in Experiment 3 only involving temporal flanker task trials. Data are shown as a function of Congruency Level of Current Trial, Congruency Level of Preceding Trial, Sequence of Target/Distractor Category (alternation, repetition), and Preceding Trial’s Congruency Level Repetition Type (alternation, repetition)

**Fig. 11:**
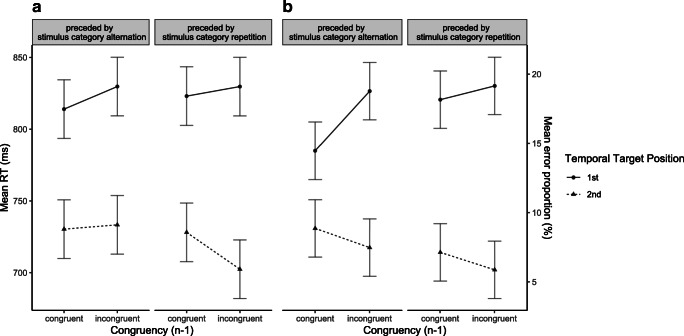
Reaction times (RTs) (**A**) and mean error proportions (**B**) of search task trials in Experiment 3. Data are shown as a function of Temporal Target Position, Congruency Level of Preceding Trial, and Preceding Sequence of Target/Distractor Category (alternation, repetition)

## Appendix A

### Results of Experiment 1 including all feature repetitions

**Table 1:** ANOVA results (one-tailed) for the mean reaction times (RTs; and for the mean error proportions in parentheses) of Experiment 1 involving all feature repetitions and repetitions of the assigned response

Predictor	*df*_*Num*_	*df*_*Den*_	*F*	*p*	*η*_p_^2^
Congruency Level of Current Trial (C)	1	15	20.04 (0.07)	< .001 (.20)	.57 (.00)
Congruency Level of Preceding Trial (C_n-1_)	1	15	8.86 (5.66)	< .001 (.025)	.37 (.27)
Sequence of Target/Distractor Category (S)	1	15	53.06 (2.73)	< .001 (.040)	.78 (.15)
C × C_n-1_	1	15	4.45 (0.33)	.009 (.17)	.23 (.02)
C × S	1	15	3.71 (.013)	.015 (.25)	.20 (.00)
C_n-1 ×_ S	1	15	1.95 (3.12)	.024 (.013)	.12 (.17)
C × C_n-1 ×_ S	1	15	1.16 (0.02)	.065 (.23)	.07 (.01)

## Appendix B

### Results of Experiment 2 including all feature repetitions

**Table 2:** ANOVA results (one-tailed) for the mean reaction times (RTs; and for the mean error proportions in parentheses) of Experiment 2 involving all feature repetitions and repetitions of the assigned response

Predictor	*df*_*Num*_	*df*_*Den*_	*F*	*p*	*η*_p_^2^
Congruency Level of Current Trial (C)	1	31	7.29 (0.99)	.055 (.017)	.19 (.031)
Congruency Level of Preceding Trial (C_n-1_)	1	31	13.80 (1.15)	< .001 (.15)	.31 (.036)
Sequence of Target/Distractor Category (S)	1	31	47.69 (0.96)	< .001 (.17)	.61 (.030)
C × C_n-1_	1	31	17.53 (0.27)	< .001 (.31)	.36 (.01)
C × S	1	31	3.4 (0.067)	.075 (.4)	.099 (.00)
C_n-1 ×_ S	1	31	0.48 (0.34)	.50 (.56)	.015 (.01)
C × C_n-1 ×_ S	1	31	6.65 (0.086)	.015 (.77)	.18 (.00)

## Appendix C

Figures and a table of the results of Experiment 3 only involving temporal flanker task trials with the added factor Preceding Trial’s Congruency Level Repetition Type (repetition vs. alternation) for reaction times and error proportions

**Table 3:** ANOVA results (one-tailed) for the mean reaction times (RTs; and for the mean error proportions in parentheses) of Experiment 3 only involving temporal flanker task trials with the added factor Preceding Trial’s Congruency Level Repetition Type

Predictor	*df*_*Num*_	*df*_*Den*_	*F*	*p*	η^2^_p_
Congruency Level of Current Trial (C)	1	22	41.73 (19.56)	< .001 (< .001)	.65 (.47)
Congruency Level of Preceding Trial (C_n-1_)	1	22	.65 (2.24)	.21 (.07)	.02 (.09)
Sequence of Target/Distractor Category (S)	1	22	3.71 (.25)	.03 (.31)	.14 (.01)
Preceding Trial’s Congruency Level Repetition Type (P)	1	22	.25 (.00)	.31 (.49)	.01 (.00)
C × C_n-1_	1	22	6.00 (1.78)	.011 (.10)	.21 (.08)
C × S	1	22	.41 (4.55)	.26 (.02)	.01 (.17)
C_n-1 ×_ S	1	22	1.12 (4.10)	.15 (.03)	.05 (.16)
C × P	1	22	.95 (1.88)	.17 (.09)	.04 (.08)
C_n-1_ × P	1	22	2.95 (.37)	.05 (.27)	.12 (.02)
S × P	1	22	.95 (.65)	.17 (.21)	.04 (.03)
C × C_n-1 ×_ S	1	22	2.18 (1.00)	.07 (.16)	.09 (.04)
C × C_n-1 ×_ P	1	22	.011 (1.64)	.46 (.11)	.00 (.07)
C × S × P	1	22	.36 (1.25)	.28 (.14)	.02 (.05)
C_n-1 ×_ S × P	1	22	.048 (1.49)	.41 (.11)	.00 (.06)
C × C_n-1 ×_ S × P	1	22	.21 (.54)	.32 (.24)	.01 (.02)

**Discussion of the above results**

As neither the three-way interaction of Congruency Level of Current Trial, Congruency Level of Preceding Trial, and Preceding Trial’s Congruency Level Repetition Type, nor the four-way interaction involving all factors reached statistical significance, our results provide no further support for Erb and Aschenbrenner’s (2019) conjecture that expectations are formed concerning the repetition of the preceding sequence of congruency levels. Although statistical power may have been insufficient to detect such subtle effects, it is also conceivable that procedural differences like the uncertainty regarding target and distractor categories (digit, letter) or the intermixed trials of the search task interfered with such expectancy formation in our experiment.

## Appendix D

Figure and table of the results of Experiment 3 for the search task trials with the added factor Preceding Sequence of Target/Distractor Category (repetition, alternation) for reaction times and error proportions

**Table 4:** ANOVA results (one-tailed) for the mean reaction times (RTs; and for the mean error proportions in parentheses) of Experiment 3 for search task trials with the added factor Preceding Sequence of Target/Distractor Category

Predictor	*df*_*Num*_	*df*_*Den*_	*F*	*p*	η^2^_p_
Temporal Target Position (T)	1	22	7.99 (18.34)	.005 (< .001)	.27 (.46)
Congruency Level of Preceding Trial (Cn-1)	1	22	.0001 (.55)	.50 (.23)	.00 (.02)
Preceding Sequence of Target/Distractor Category (S_n-1_)	1	22	.51 (.03)	.24 (.43)	.02 (.00)
T × C_n-1_	1	22	.90 (4.16)	.18 (.03)	.04 (.16)
T × S_n-1_	1	22	1.04 (2.85)	.16 (.05)	.05 (.11)
C_n-1_ × S_n-1_	1	22	.83 (.59)	.19 (.22)	.04 (.03)
T × C_n-1_ × S_n-1_	1	22	.25 (.74)	.31 (.20)	.01 (.03)

## Footnotes

1 In fact, asking participants to alternate between executing a digit identification task and a letter identification task while presenting digit-letter pairs is a frequently used procedure in task switching experiments (e.g., Brown et al., 2007; Rogers & Monsell, 1995).

2 We thank an anonymous reviewer for encouraging us to run a replication experiment.

3 It must even be considered that different types of repetition effects pertain to first- and higher-order repetitions of the congruency level. Noteworthy in this connection, Erb and Aschenbrenner (2019), using the split-task method, found the CSE selectively in sequences in which the congruency level of trial N-1 matched the level of trial N-2. The authors accounted for this result pattern assuming expectation of a repetition of the previous congruency level as well as of repetition of the previous congruency level sequence (i.e., repetition vs. alternation), acknowledging, however, that “N-2-to-N” feature sequences might provide an alternative explanation.

4 Additional analyses, including the factor “Preceding Sequence of Target/Distractor Category (repetition vs. alternation)” of the preceding temporal flanker task trial yielded no indication that the search task effect differed after repetition trials compared to alternation trials (*F*(1, 22) = .25, *p* = .31, *η*_*p*_^2^ = .01; *F*(1, 22) = .74, *p* = .20, *η*_*p*_^2^ = .04) for RTs and errors respectively. See Appendix D for a detailed description of the results of these analyses.
